# Structural and Functional Heat Stress Responses of Chloroplasts of *Arabidopsis thaliana*

**DOI:** 10.3390/genes11060650

**Published:** 2020-06-12

**Authors:** Puneet Paul, Anida Mesihovic, Palak Chaturvedi, Arindam Ghatak, Wolfram Weckwerth, Maik Böhmer, Enrico Schleiff

**Affiliations:** 1 Department of Biosciences, Molecular Cell Biology of Plants, Goethe University, 60438 Frankfurt am Main, Germany; puneet6288@gmail.com (P.P.); anida.mesihovic@gmail.com (A.M.); Boehmer@bio.uni-frankfurt.de (M.B.); 2 Molecular Systems Biology (MOSYS), Department of Functional and Evolutionary Ecology, University of Vienna, 1090 Vienna, Austria; palak.chaturvedi@univie.ac.at (P.C.); arindam.ghatak@univie.ac.at (A.G.); wolfram.weckwerth@univie.ac.at (W.W.); 3 Vienna Metabolomics Center (VIME), University of Vienna, 1090 Vienna, Austria; 4 Frankfurt Institute of Advanced Studies (FIAS), D-60438 Frankfurt am Main, Germany

**Keywords:** chloroplasts, proteome, heat stress, translocon, ppi1, toc64, quantitative proteomics

## Abstract

Temperature elevations constitute a major threat to plant performance. In recent years, much was learned about the general molecular mode of heat stress reaction of plants. The current research focuses on the integration of the knowledge into more global networks, including the reactions of cellular compartments. For instance, chloroplast function is central for plant growth and survival, and the performance of chloroplasts is tightly linked to the general status of the cell and vice versa. We examined the changes in photosynthesis, chloroplast morphology and proteomic composition posed in *Arabidopsis thaliana* chloroplasts after a single or repetitive heat stress treatment over a period of two weeks. We observed that the acclimation is potent in the case of repetitive application of heat stress, while a single stress results in lasting alterations. Moreover, the physiological capacity and its adjustment are dependent on the efficiency of the protein translocation process as judged from the analysis of mutants of the two receptor units of the chloroplast translocon, TOC64, and TOC33. In response to repetitive heat stress, plants without TOC33 accumulate Hsp70 proteins and plants without TOC64 have a higher content of proteins involved in thylakoid structure determination when compared to wild-type plants.

## 1. Introduction

The maintenance of cellular homeostasis depends on a multitude of cellular processes, which ensures cellular and organismic survival [1]. Among others, the balancing and reprogramming of protein levels and composition is essential, which includes protein synthesis, folding, turnover and maintenance [2,3,4]. Equally important is the proper distribution of proteins within cells and their insertion into the destined compartments [5,6,7,8], which is either mediated by specialized translocon components within membranes or by vesicle transport [9,10]. Today, it is established that specific mechanisms that control the functional state of an organelle are interlinked with these translocation paths [11,12,13,14]. On the one hand, this is of particular importance considering that organelles such as chloroplasts employ thousands of different proteins synthesized in the cytosol to perform fundamental processes such as photosynthesis, amino acid, and lipid biosynthesis [15]. On the other hand, protein homeostasis and distribution in plant cells is sensitive to environmental changes [16,17,18]. For instance, temperature changes are known to affect multiple aspects of cellular homeostasis, including the regulation and distribution of proteins in cellular sub-compartments [19,20]. In general, the effect of heat stress (HS) on the proteome of whole tissues or organs has been well documented [21,22,23,24,25]. In addition, first insights were obtained into the organellar proteins in response to the HS of chloroplasts [19,26].

Chloroplasts are semi-autonomous organelles of endosymbiotic origin, and most chloroplast proteins are synthesized in the cytosol and transported across the chloroplast envelope membranes [27]. The general effect of HS on the functional properties of chloroplasts is well established [28,29,30]. For example, the inhibition of the carbon assimilation system [31], the over-reduction of the plastidic electron transport chain that results in enhanced reactive oxygen species (ROS) production [26,32], as well as the reduction of Rubisco activase activity [31], all lead to a reduction in photosynthesis under stress condition [33]. The functional adaptation is accompanied by changes in the morphological features of chloroplasts. It has been established that HS induces changes in the macroscopic structure of chloroplasts [34,35] and the reorganization of thylakoid membranes [36]. However, the effects of short and repetitive HS have not been documented for the structure and function of the chloroplasts.

Furthermore, it has been shown that translocation capacity and complex composition of the chloroplasts translocon is negatively affected upon HS exposure [37]. Moreover, chloroplasts’ precursor proteins accumulate in the cytosol upon HS [38]. This indicates the involvement of chaperones in protein transport to the surface of the chloroplast [39]. Thus, HS appears to affect the protein translocation event and indirectly modulate the translocation efficiency by pre-protein aggregation, or at least accumulation in the cytoplasm.

The translocon of the outer membrane of the chloroplasts (TOC) has four central components [5,6,9,12,27]. While three form the so-called core complex for translocation providing the final precursor protein receptor sides and the translocon [5,6], the fourth component, TOC64 (TOC component of 64 kDa), is discussed to provide a chaperone platform [40,41,42,43]. Its impact on protein translocation is regulatory, as a mutant lacking TOC64 function does not show a significant reduction of protein translocation efficiency on its own [44,45], but in conjunction with a mutant of the precursor protein receptor component TOC33 (TRANSLOCASE OF CHLOROPLAST 33) of the TOC core complex annotated as *ppi1* [43]. However, the importance of the chaperone system for HS response reaction [46,47,48] renders TOC64 an exciting candidate for the integration of HS regulation and protein translocation. The interaction of TOC64 with chaperones depends on a so-called tetratricopeptide repeat (TPR) [41,49,50]. This domain recognizes the C-terminus of HSP90 (HEAT SHOCK PROTEIN 90) and HSP70 (HEAT SHOCK PROTEIN 70) chaperones [41] with a dissociation constant of about 200 µM [51], while the dissociation constant of the interaction between full-length TOC64 and full-length chaperones is in the range of about 2-15 µM [52]. The regulation of this interaction might depend on the recognition of auxin derivatives [42] or phosphorylation as established for the mitochondrial localized paralogue [53]. The latter, however, has not yet been established for the chloroplast localized form.

Here, we investigated the effect of HS on chloroplasts structure and function. For this, we considered wild-type *Arabidopsis thaliana* (Col-0) plants (WT), a *toc64-III* mutant [43] and a mutant of the TOC core complex component TOC33, *ppi1* [54]. The plants were exposed to HS either once (HS-I; single term) or repeatedly (HS-II; repetitive). The latter was considered foreseeing the frequent episodes of HS in nature [55]. We observed that physiological parameters, including chlorophyll content and photosynthetic activity as well as chloroplasts ultrastructure, were altered in the mutants compared to wild-type plants under control conditions as well as under the two HS regimes. Moreover, significant alterations in the proteomes of mutants and wild-type plants were detected under control as well as under different heat stress regimes. Considering these obtained results, both WT and the mutant plant lines exposed to regular episodes of HS (HS-II) performed better compared to the plants exposed to HS only once (HS-I), thereby signifying that plants modulate structural and functional features of chloroplasts to acclimatize under regular episodes of HS.

## 2. Materials and Methods

### 2.1. Plant Material and Stress Treatment

**Figure 1:**
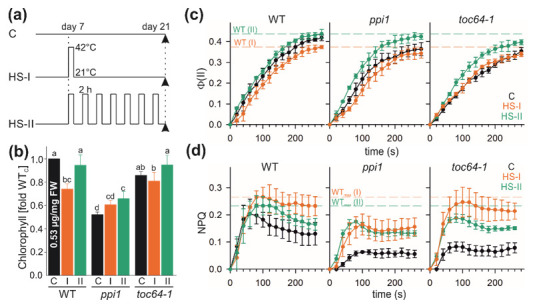
Chlorophyll content and photosynthetic performance after heat stress. (**a**) Schematic representation of the heat stress (HS) regime. *A. thaliana* wild type, *ppi1*, and *toc64-1* were grown under short-day conditions and were then either kept under control (**c**) conditions (21 °C) or submitted to heat stress conditions (42 °C) either only once on day 7 for 2 h (HS-I) or every alternate day (HS-II) until day 21. (**b**) Chlorophyll content from 21-day-old leaves of wild-type, *ppi1*, and *toc64-1* plants subjected to control (**c**), HS-I (I), or HS-II (II) treatment as shown in (**a**). The values shown are the means of at least three measurements (± SD). Statistical analysis (see methods section) was performed (*p* < 0.05). (**c**,**d**) The kinetic of the quantum yield adaptation of photosystem II (**c**) or the non-photochemical quenching (NPQ; **d**) from 21-day-old leaves of wild-type (left), *ppi1* (middle) or *toc64-1* plants (right) after they have been subjected to control (black), HS-I (orange), or HS-II treatment (green) at low light conditions is shown. The maximal Φ(II) and NPQ value for HS-treated wild-type plants is indicated as a horizontal dashed line for comparison.

*A. thaliana* wild-type (Col-0), *ppi1*, and *toc64-III-1* plants [43,55] were grown for one week under an 8/16 h day/night cycle (21/18 °C). All cotyledons were fully opened. Plants were either kept under control conditions for the next 14 days (C), exposed to a single heat-stress on day 7 (HS-I: 42 °C for 2 h) and subsequently incubated as under control conditions for 14 days (single HS treatment), or every alternate day from day 7 onwards for the next 14 days (HS-II: 42 °C for 2 h; repetitive/periodic/repeatedly HS, Figure 1). After the treatment, all plants had > 6 rosette leaves and > 20% of the final rosette diameter, but inflorescence did not yet emerge. Plants treated with the respective conditions were used to analyze physiological parameters and proteomics at the time point of harvesting.

### 2.2. Measurement of Chlorophyll and Photosynthetic Activity

The measurement of chlorophyll and photosynthetic activity (actinic light: 134 µmol m**^−^**^2^ s**^−^**^1^) of wild-type and mutant plant lines was performed as defined before [43,56]. For measuring chlorophyll content, at least three plants (biological replicas) were used to calculate means (± SD) and statistical analysis was done using analysis of variance (ANOVA) and two-sided Student’s *t*-test. For the quantum yield, at least four plants (two biological replicas) with at least two areas of interest each were used to calculate means (± SD). Furthermore, a two-sided dependent samples *t*-test was used to compare the pairs of time points between two conditions or genotypes.

### 2.3. Analysis of Chloroplast Ultrastructure by Transmission Electron Microscopy

The leaves of wild-type and mutant plants were harvested, cut into 2–3-mm pieces, and fixed in 4% (v/v) glutaraldehyde in 50 mM Na-cacodylate buffer pH 7.4 for 2 h at room temperature. After washing, the ‘post-fixation step’ was performed in 1% (w/v) OsO_4_ in the same buffer at room temperature for 2 h. The samples were washed and dehydrated in a graded series of aqueous ethanol solutions (30–90%, v/v) followed by three changes in 100% ethanol and two changes in propylene oxide for 30 **min** at each step. Afterwards, samples were infiltrated in a graded series of epoxy resin (Araldite CY212, Agar Scientific, Stansted, UK) in propylene oxide- ratio 2:1 and 1:1 (v/v), each for 2 h. The samples were then transferred through two changes of 100% embedding medium, each for 3 h. The samples were kept in fresh 100% embedding medium, and the polymerization of specimens was performed in molds at 65 °C for 48 h. Ultrathin (50 nm) sections were made using a diamond knife with ultra-microtome and collected on copper grids. The sections were stained in 2% *w/v* uranyl acetate for 15 min, followed by 10 min of staining in Reynold’s lead citrate [56,57]. Transmission electron micrographs were taken on a Philips CM12 Transmission Electron Microscope operated at an accelerating voltage of 80 kV. Digital images were obtained using a Gatan Erlangshen ES500W (model 782) CCD camera (Pleasanton, CA, USA).

### 2.4. Isolation of Chloroplasts

The isolation of chloroplasts was performed as previously described [58]. Briefly, 21-day-old *A. thaliana* plants (WT and mutants) corresponding to each of the temperature regime (control, HS-I, and HS-II) were dark-adapted for half an hour before chloroplasts isolation. The isolation procedure was carried out at 4 °C. Three biological replicates of leaves from of 50 plant each were processed. Leaves were pooled and grounded using Ultra Turrax homogenizer in the isolation buffer (Sorbitol: 450 mM, Tricin: 20 mM and pH 8.4, magnesium chloride hexahydrate: 5 mM, EDTA: 10 mM, sodium bicarbonate: 5 mM). The homogenate was then passed through filtrate and centrifuged at 1500× *g* for 5 min. The pellet thus obtained was re-suspended in wash buffer (Sorbitol: 300 mM, Tricin: 20 mM and pH 7.6; magnesium chloride hexahydrate: 5 mM, EDTA: 2.5 mM). The suspended pellet was loaded onto a percoll gradient (top: 45% and bottom: 85%; Sorbitol: 300 mM, Tricin: 20 mM and pH 7.6; magnesium chloride hexahydrate: 5 mM and percoll) and spun at 10,000× *g* for 10 min. Then, the topmost layer was removed using the suction pressure to obtain middle green layer having intact chloroplasts. The intact chloroplasts were washed twice with the wash buffer and the pellet was finally suspended in 300 µL wash buffer. For protein extraction, isolated chloroplasts were dissolved in sodium-dodecyl-sulfate (SDS) buffer, and protein concentration of the samples was measured using amido black [59].

### 2.5. Shotgun Proteomics (GEL-LC-Orbitrap-MS)

Proteomics analysis of the isolated chloroplasts was performed as described before [60,61]. Pre-fractionation of the proteins was carried out by SDS-PAGE [60]. Fourty micrograms of total protein was loaded onto a gel and run for 1.5 cm. The gels were fixed and stained with in methanol:acetic acid:water:Coomassie brilliant blue R-250 (40:10:50:0.001), destained in methanol:water (40:60) and then each lane was divided into two fractions. Furthermore, the gel pieces were destained, equilibrated and digested with trypsin, desalted and concentrated according to [60]. A total of 10 μg of digested protein was injected (three technical replicates) into a one-dimensional (1D) nano-flow LC–MS/MS system equipped with a pre-column (C18, Eksigent, Redwood City, CA, USA). Peptides were eluted using a Ascentis column (Ascentis Express, peptide ES-C18 HPLC column (SUPELCO Analytical, Bellefonte, PA, USA), dimension 15 cm × 100 μm, pore size 2.7 μm) during an 80 min gradient from 5% to 50% (v/v) acetonitrile, 0.1% (v/v) formic acid. MS analysis was performed on an Orbitrap LTQ XL mass spectrometer (Thermo, Dreieich, Germany) with a controlled flow rate of 500 nl per minute. The specific tune settings for the MS were as follows: spray voltage was set to 1.8 kV; temperature of the heated transfer capillary was set to 180 °C. Each full MS scan was followed by ten MS/MS scans, in which the ten most abundant peptide molecular ions were dynamically selected, with a dynamic exclusion window set to 90 s. Dependent fragmentation was performed in collision-induced dissociation (CID) mode, with a normalized collision energy of 35, an isolation window of 1.0, an activation Q of 0.250 and a 30-ms activation time. Ions with a + 1 or unidentified charge state in the full MS were omitted from MS/MS analysis.

### 2.6. Quantitative Analysis of the Proteome

Label-free quantification (LFQ) of proteins was performed as previously described [62]. Acquired spectra were processed using MaxQuant version 1.6.12.0 [63]. For database searches against the Araport 11 database, the precursor mass tolerance was set to 20 ppm for the first search and 4.5 ppm for the main searches. Trypsin/P was chosen as the enzyme with two missed cleavages allowed. Oxidation of methionine and protein N-terminal acetylation were defined as variable modifications, and carbamidomethylation of cysteine was defined as a fixed modification. The minimum peptide length was set to seven amino acids. The LFQ intensities for the detected protein groups were used and zero values adjusted to the lowest LFQ value (> 0) to determine the minimal fold change of abundance. Further details are in the text. For the functional assignment, we used MapMan [64] and TAIR10 [65]. If not otherwise noted, protein localization was extracted from the subcellular localisation database for Arabidopsis proteins (SUBA) [66]. The data for Table 1 and Table 2 were processed with custom R scripts (Supplemental File S1) and manually filtered for proteins localized to chloroplasts (see Supplementary Material). All graphs were processed with SigmaPlot, and further information was extracted from the literature.

## 3. Results

### 3.1. Heat Stress Alters Chlorophyll Content and Photosynthetic Performance

Previous studies inspecting the impact of heat stress (HS) on the performance of chloroplasts have focused either on a single or repetitive HS treatment [19,26,37,67]. However, considering the current climate change forecasts [55], we designed an experimental set-up where plants, after the initial germination and growth for 7 days, were treated with HS either once (HS-I, Figure 1a) or every alternate day until day 21 (HS-II). All three *A. thaliana* lines, wild type (*A. thaliana* Col-0)*, ppi1*, and *toc64-III-1* (*toc64-1* hereafter) were exposed to these conditions.

As previously established, the wild-type plants grown under control conditions showed the highest chlorophyll content, while *ppi1* displayed the lowest (Figure 1b, [43]). The latter is consistent with the pale phenotype of *ppi1* [54]. After single HS (HS-I), chlorophyll content in the wild type was significantly reduced when compared to untreated plants (Figure 1b). In contrast, HS-I application yielded no significant change of the chlorophyll content in *ppi1* or *toc64-1* when compared to control conditions (Figure 1b). Remarkably, the chlorophyll content after the repetitive HS treatment (HS-II) of plants, irrespective of the genotype, was higher relative to HS-I (Figure 1b). In wild-type and *toc64-1* plants, the chlorophyll content after the repetitive HS treatment (HS-II) reaches the levels of untreated wild-type plants, while in *ppi1* plants it is significantly higher than in untreated plants.

We confirmed that both untreated mutants have a lower effective Photosystem II (PSII) quantum yield (Φ(II)) when compared to untreated wild-type plans (Figure 1c, black symbols). Moreover, we observed a higher quantum yield for all plants exposed to HS-II when compared to the plants exposed to HS-I (Figure 1c). However, in wild-type plants, the quantum yield after HS-II is comparable to the one observed for untreated plants, while, in both mutants, the quantum yield after HS-I is more comparable the one observed for the respective untreated genotype (Figure 1c).

We further analyzed non-photochemical quenching (NPQ) as a measure of the capacity of the plants to regulate and by that to protect photosystems when light absorption exceeds the capacity for light utilization [68]. For wild-type plants grown under normal conditions, NPQ rapidly rises to a maximum of the NPQ (Figure 1d, left, black symbols). Subsequently, the NPQ gradually decreases, which suggests that the photosystem adapts to high light conditions (Figure 1d, left, black symbols). Both mutants show a reduced NPQ, which indicates that the capacity to regulate the photosystems is reduced (Figure 1d, black symbols). After HS, the NPQ is enhanced in wild-type when compared to untreated plants, which can be interpreted as an adaptation to the stress condition (Figure 1d, left green and orange symbols). The two mutant lines also show an enhanced NPQ after HS treatment when compared to the respective genotype without treatment (Figure 1d). This suggests that HS induces the accumulation of protective molecules. However, *ppi1* does not reach the maximal levels of the NPQ of wild-type plants after HS-I, while *toc64-1* shows a comparable behavior of NPQ to the wild type (Figure 1d, orange symbols). In turn, after HS-II, both mutants do not reach the maximal NPQ level of the wild-type plants, while the steady-state level is comparable (Figure 1d, green symbols). Hence, while the chlorophyll content and the effective PSII quantum yield and the NPQ appear to be enhanced after HS-II in all genotypes, the NPQ in the mutants does not reach the wild-type level after this stress.

### 3.2. The Impact of Heat Stress on Chloroplasts Structure

**Figure 2:**
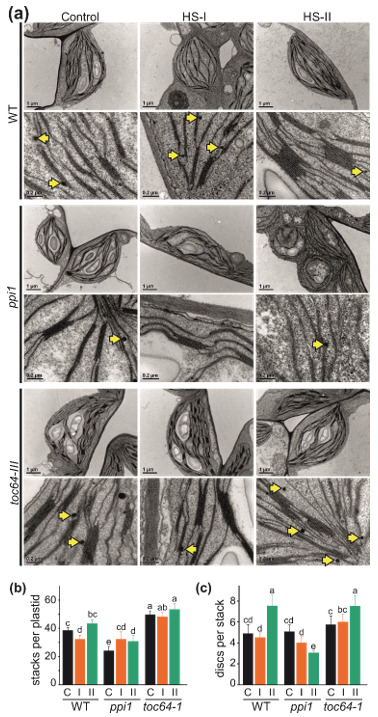
Chloroplast ultrastructure of heat-stress-treated wild-type, *ppi1*, and *toc64-1* plants. (**a**) The leaves of 21-day-old plants were analyzed. Representative images of each line and condition are shown in two magnifications (11,500×: complete chloroplast and 66,000×: for stacks). Scale bar for each image shows 1 µm for the 11,500× fold magnification and 0.2 µm for 66,000× fold magnification. Plastoglobules are marked by yellow arrows. (**b**,**c**) The number of grana stacks and discs per grana stack was counted for each plant line (at least four chloroplasts per plant line per condition). Significance (*p* < 0.05; n = 5) determined by statistical analysis (ANOVA with Duncan post hoc test) is indicated.

The observed alterations of chlorophyll content and photosynthetic performance prompted the investigation of the ultrastructure of the chloroplasts (Figure 2a). Consistent with the previous studies [54,56], a reduced number of stacks per plastid were observed for *ppi1* compared to wild-type plants under control conditions (Figure 2a,b, black bar). In contrast, the number of stacks is significantly higher in chloroplasts of *toc64-1* leaves (Figure 2a,b, black bar). When plants were exposed to a single heat stress (HS-I), the number of stacks per chloroplasts is reduced in wild-type, not significantly altered in *toc64-1*, but enhanced in *ppi1* to the number seen in wild-type for the particular condition (Figure 2a,b, orange bar). On the other hand, after repetitive HS treatment (HS-II), the stack number in wild-type and *toc64-1* is similar to the chloroplasts from untreated plants, while the number is higher in *ppi1* relative to untreated plants but lower than wild-type plants in HS-II (Figure 2a,b, green bar).

The number of stacks per disc is not significantly different between the three genotypes grown under standard conditions as well as HS-I treatment (Figure 2a,c, black and orange bar). After HS-II, we observed an increase of the discs per stack in wild-type and *toc64-1*, while the number is reduced in *ppi1* (Figure 2a,c, green bar). It is further noticeable that the thylakoids of chloroplasts of *ppi1* are billowy after application of HS-I (Figure 2a). The same is observed for chloroplasts of *toc64-1* grown under control conditions or after HS-I, but not after HS-II (Figure 2a). Moreover, we realized an accumulation of plastoglobuli in this line after HS-II treatment (Figure 2a, yellow arrow).

### 3.3. The Chloroplast Proteome of Wild-Type, ppi1, and toc64-1

**Figure 3:**
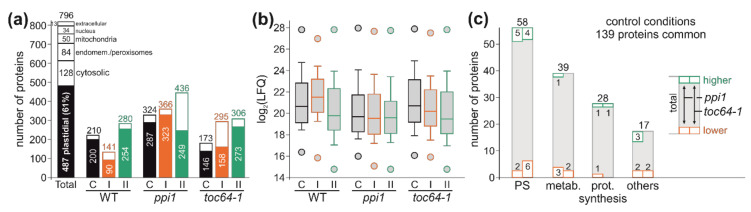
Proteome analysis of chloroplasts. (**a**) The number of identified proteins is shown. “Total” indicates all proteins identified in at least one sample; subsequently, the number of proteins discovered in individual samples is shown (black: control, orange: HS-I, green HS-II). The full bar indicates the fraction of plastid localized proteins based on SUBA, and the open segment indicates the fraction of proteins with localization to other cellular sub-compartments. The total number of discovered proteins is indicated on top. (**b**) The box plot of the log_2_(LFQ) value distribution with indicated maximal and minimal values is shown for each fraction. (**c**) The proteins detected in all three genotypes under control conditions are compared. The total number of proteins involved in photosynthesis (PS), metabolism, protein synthesis, and other process is shown, and proteins with lower (red) or higher (green) abundance in the mutants relative to wild-type plants are shown as indicated on the right.

Having assessed the physiological and morphological changes of chloroplasts after exposure to different HS regimes (Figure 1 and Figure 2), we analyzed their proteomic regulation. The proteomic analysis of all samples (3 genotypes and 3 growth conditions) yielded a total of about 800 different proteins considering proteins with a label-free quantification (LFQ) value > 0 in at least one condition (Figure 3a, Tables S1 and S2). According to data from SUBA [66], about 60% of the proteins are assigned as plastid localized (Figure 3a). Within the individual chloroplast fractions corresponding to different genotypes and treatments, the number of identified proteins varied between 140 and 440 with the lowest number of identifications in the wild-type background after HS-I (Figure 3a). The discovery of proteins assigned as plastid localized ranges from 90 to 323 (Figure 3a). The wide distribution of the LFQ values in different fractions justified a further analysis (Figure 3b). Only the values for the wild-type chloroplasts after HS-I might be overrated to a certain extent (Figure 3b).

At first, we analyzed the overlap of the untreated samples from different genotypes. We identified 152 proteins represented by 136 protein groups in all three control fractions. In total, 139 proteins (130 protein groups) thereof were assigned as plastid localized. The plastid localized proteins can be categorized as proteins involved in metabolic processes, protein synthesis (including transcription, translation, synthesis, and degradation), photosynthesis (photosystems, light-harvesting complexes (LHCs), photosystem assembly, and regulation) and others (general regulation, membrane proteins, and proteins of unknown functions; Figure 3c). Comparing the proteins found in all three genotypes, only a few exhibited higher (green) or lower abundance (orange) in the mutant lines (Figure 3c, Table 1). In *ppi1,* we found 18 proteins to be altered at least two-fold in their abundance and in *toc64*-*1*, 15 proteins with at least a two-fold change in abundance were detected relative to wild-type plants. The low number of proteins with altered abundance is expected as the *ppi1* phenotype is most prevalent in the early growth stages [54,56] and the phenotype of *toc64-1* is generally not very strong when plants are grown under standard conditions [41,43,44,45]. Two proteins showed a less abundance in both mutants (Table 1). The 2-Cys PrxB (AT5G06290; Table 1) is involved in the pathway for energy dissipation in photosynthesis and peroxide detoxification in the dark [69], and its functionality is related to the NPQ efficiency [70]. The thioredoxin M-type 1 (Trx-m1, AT1G03680, Table 1), downregulated in *ppi1*, is discussed to regenerate 2-Cys PrxB in the light [71] and to be involved in the biogenesis of PSII [72]. The second protein less abundant in both mutants is a component of the light-harvesting complex I, LHCa4 (AT3G47470, Table 1). However, LHCa1 (At3g54890) and LHCa2 (AT3G61470) were detected in all three chloroplast fractions as well but without drastic alterations between the mutant plant lines and wild-type plants. Only LHCa3 (AT1G61520) abundance is reduced in chloroplasts isolated from *toc64-1* leaves, but not in chloroplasts isolated from the *ppi1* background.

Only one protein, a component of the plastidic ribosome, is enhanced in the chloroplasts of the two mutants. Moreover, we found six proteins in *toc64-1* and *ppi1*, but not in the wild type. HHL1 (AT1G67700) is involved in PSII protection from photo-damage [73], and RBD1 (AT1G54500) is required for establishing PSII function [74]. Moreover, Lil3.1 (AT4G17600) is described to be involved in the regulation of late events of chlorophyll biosynthesis [75]. Besides, the nitrite reductase NIR1 (AT2G15620), a protein likely acting as thioredoxin (AT5G03880) and an HSP70 family protein with plastidic localization (AT1G56410) were found in the chloroplasts of the two mutants, but not in wild-type chloroplasts.

### 3.4. Proteome Changes in Response to Heat Stress

**Figure 4:**
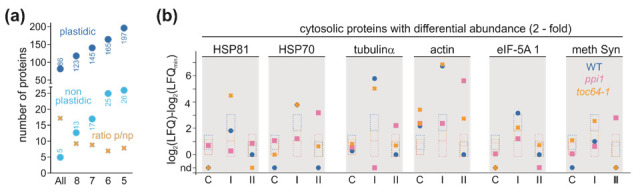
Protein abundance of non-plastidic proteins in chloroplast fractions after heat stress. (**a**) The number of plastid (p -localized (dark blue) or non-plastidic (np) proteins (light blue; based on SUBA) found in all, or in at least 8, 7, 6 or 5 chloroplast samples is shown. Yellow crosses indicated the ratio between plastid localized proteins and non-plastidic proteins. (**b**) The log_2_(LFQ) for the indicated protein group was normalized to the lowest log_2_(LFQ) value found for the indicated group in any of the samples. The value is plotted for the wild type (blue), *ppi1* (magenta) or *toc64-1* (yellow). The dotted frames indicate the 99% confidence interval of the median of the log_2_(LFQ) values normalized to the lowest median (control, HS-I, and HS-II) of all non-plastidic proteins in one sample.

Focusing on the entire data set, we used only the proteins that were discovered in at least five independent experiments with an LFQ > 0 (Figure 4a). This number considers that proteins might only become abundant and detectable after heat treatment allowing one false negative discovery (FDR). According to the above-formulated criteria, 26 non-plastidic proteins corresponding to 16 protein groups were identified (Figure 4a). Seven of these groups are components of the endomembrane system, including vacuoles and peroxisomes, two are mitochondrial localized proteins, and one is a nuclear protein. These proteins are either highly abundant in cells (e.g., the Catalase 3, AT1G20620; the peroxisomal NAD-malate dehydrogenase 2, AT5G09660 or the lactate/malate dehydrogenase, AT1G53240 and AT3G15020) or integral membrane proteins (e.g., vacuolar ATP synthase subunit A, AT1G78900 or the water and ammonium transporter, AT3G16240). Thus, although it cannot be excluded that these proteins are dual localized, they are most likely contaminations resulting from the isolation procedure.

The remaining six groups constitute cytosolic proteins: HSP81, HSP70, actin, tubulin, methionine synthase, and translation initiation factor protein families. For the analysis of the abundance variation, we compared the abundance of the individual protein groups in one sample to the median of the abundance of all non-plastidic proteins considering that most of them are likely contaminants. HSP70 is accumulated in the chloroplast fraction of *toc64-1* plants and wild-type plants after HS-I, and of *ppi1* after repetitive HS treatment (HS-II, Figure 4b). Moreover, HSP81 exhibits higher abundance in the chloroplast sample of *toc64-1* plants and wild-type plants after HS-I (Figure 4b). Tubulin-α shows a similar distribution as seen for HSP70, while actin appears to be generally more abundant (Figure 4b). In contrast, the identified eukaryotic translation initiation factor 5A-1 (AT1G26630) protein does not show an enrichment, and the methionine synthase (AT5G17920 and AT3G03780) is only higher more abundant than average in chloroplasts of *ppi1* plants after HS-II (Figure 4b).

Next, we analyzed the plastidic proteins. To estimate the error of the change, we used all 197 plastidic proteins found in at least five independent discoveries, normalized the log_2_(LFQ) values for a given protein to the lowest log_2_(LFQ) value found for that protein and determined the median of the change with a 99% confidence interval value. From all observed values, the median was determined, resulting in 0.85 as the median of all changes and 0.7 as the limit for the 99% confidence of alteration from the median. Thus, for the subsequent analysis, only changes between two samples of at least Δlog_2_(LFQ) > 1.65 were discussed. Moreover, we determined the distribution of the log_2_(LFQ)-log_2_(LFQ)min of the selected proteins for each chloroplast fraction to judge whether the distribution is comparable. Statistical analysis revealed that all samples are comparable except for the wild type and *toc64-1* after HS-I, which show significantly lower change values. Hence, for these two samples, a higher false-negative rate is expected, while, in comparison to these two probes, a higher false-positive rate is expected concerning these probes. Nevertheless, this analysis shows that a call for a change of Δlog_2_(LFQ) > 1.65 is consistent with the distribution.

After realizing that the protein abundance under control conditions is somewhat comparable in the different genotypes (Figure 3), we determined which protein was either decreased or increased by Δlog_2_(LFQ) > 1.65 in response to HS in a given genotype. In case of no detection, the value was considered to be the average of the value for the same condition in the other genotypes. By the above-described procedure, we detected 28 proteins in 27 protein groups assigned as plastidic in SUBA with altered abundance in response to heat stress. Global assignment revealed that nine are involved in metabolic processes, nine in the photosynthetic processes, seven in processes related to protein synthesis, two in general regulation and one in transport processes.

**Figure 5:**
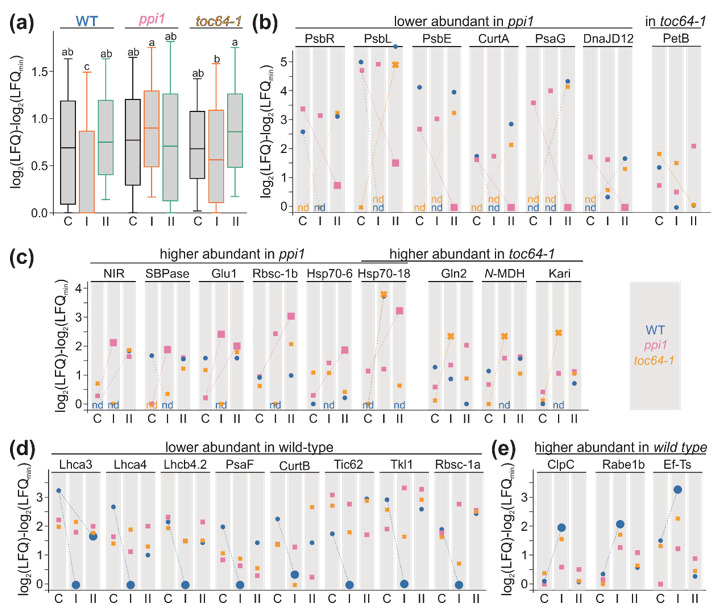
Abundance of plastidic proteins in chloroplast fractions after heat stress. (**a**) The distribution of the log_2_(LFQ) values for a protein normalized by subtracting the smallest found value was analyzed for each probe. The box plot presentation is shown. The difference in the distribution was analyzed by ANOVA. (**b**–**e**) The log_2_(LFQ)-log_2_(LFQ_min_) values are plotted for proteins with a difference > 1.65 between control, and one HS-treated sample for at least one genotype. The symbols are colored according to the legend. Enlarged symbols and dashed lines indicate changes (>1.65) after HS. Dotted lines indicated changes (>1.65), assuming that the absence of detection suggests a protein abundance lower or equal to the lowest LFQ value observed for this protein.

Three components of photosystem I, a protein involved in thylakoid structure determination (CurtA, AT4G01150 [76]), as well as PsaG and a plastid-targeted DnaJ protein (AT3G51140) are reduced in their abundance in *ppi1* after HS-II (Figure 5b). It appears that all proteins except the DnaJ protein are highly abundant in chloroplasts isolated from *toc64-1* and the wild type grown after the repetitive heat treatment (HS-II, Figure 5b), but are either not or only at low abundance detected in chloroplasts isolated from *toc64-1* plants grown under normal conditions or HS-I (Figure 5b). In contrast, PetB is reduced in its abundance in *toc64-1* after HS-II, while it is generally decreased in wild type after HS and even enhanced in *ppi1* after HS-II (Figure 5b).

In *ppi1*, we detected a higher abundance for six proteins in chloroplasts isolated from plants after HS (Figure 5c). Here, the nitrite reductase NIR1 (AT2G15620) and the sedoheptulose-bisphosphatase (SBPase, AT3G55800) were higher abundant after HS-I, and the glutamate synthase (Glu1, AT5G04140) showed higher abundance after HS-I and HS-II. The small subunit of the ribulose bisphosphate carboxylase (Rbsc-1b, AT5G38430) and two HSP70 shown (AT4G24280 [77]) or predicted to be localized in plastids (HSP70-18, AT1G56410) are enhanced in chloroplasts isolated after HS-II application. The latter is enhanced in chloroplasts of *toc64-1* plants as well, but only after HS-I (Figure 5c). In addition, we found that dual-targeted glutamine synthetase (Gln2, AT5G35630 [78]), the NADP-dependent malate dehydrogenase (*NADP-*MDH, AT5G58330 [79]) and ketol-acid reductoisomerase, necessary for amino acid biosynthesis (KARI, AT3G58610), are enhanced in chloroplasts isolated from *toc64-1* after HS-I (Figure 5c).

After inspecting the changes in protein abundance in the mutants, we analyzed the changes in the wild type. We detected eight proteins exhibiting lower abundance after HS, mostly after HS-I. While it might be in part due to false-negative discovery (see discussion above, Figure 5a), remarkably, 4/8 proteins correspond to the light-harvesting system or photosystem II (Figure 5d). Further, a reduction of these proteins is consistent with the reduced chlorophyll content and the lower photosynthetic performance after HS-I treatment (Figure 1). These proteins also showed lower abundance after HS-II, although only Lhca3 fulfils the selection criteria (LFQ > 1.65; Figure 5d). Additionally, three components of photosystem I are not detected in wild-type chloroplasts after HS-I, but in chloroplasts isolated from plants without HS (Figure 5b). A similar distribution was found for CurtA, and the other protein of this family, CurtB (AT2G46820 [76]), is found to be reduced under this condition as well (Figure 5d). Moreover, the redox regulation of protein import (Tic62, AT3G18890 [80]), carbon fixation (Rbsc-1a, AT1G67090) or thiamine pyrophosphate synthesis (TKL1, AT3G60750 [81]) are reduced in their abundance in wild-type chloroplasts after HS-I (Figure 5d). Furthermore, we found four proteins with higher abundance in wild-type chloroplasts after HS-I, namely the class I Clp chaperones ClpC (AT3G48870, AT5G50920; Figure 5e) that are required for protein quality control [81], the translation elongation factor Rabe1b (AT4G20360), and its nucleotide exchange factor Ef-Ts (AT4G29060) known to be necessary for the HS response in plastids [82] (Figure 5e). All three protein families are enriched in chloroplasts of *toc64-1* and *ppi1* after HS-I as well, but not as drastic as in the wild type.

## 4. Discussion

### 4.1. The Change of Chloroplast Proteome and Performance in Mutants of the TOC Translocon

The TOC complex is the central entry gate for protein translocation. Toc33 is a component of the TOC core complex involved in the regulation of precursor protein reception and delivery [5,6,9,12,27]. In contrast, TOC64 is only dynamically associated with the Toc complex [40,41,42,43,44,45]. The function in chaperone recognition by its cytosolic exposed TPR motif [43,49,50,51,52] places TOC64 upstream of the core complex. At the same time, an important role in chloroplast quality control is possible as precursor protein recognition by chaperones in cells regulates their abundance in chloroplasts as well [83], as the two proteins are important for chloroplast biogenesis that takes place most rapidly in developing plants. Thus, the majority of the previous studies focused on earlier growth stages [43,54,56,84]. Here, we analyzed the performance of later growth stages that are more likely prone to be exposed to heat stress. Nevertheless, our results for control conditions provide information on the importance of the two components for the steady-state performance of chloroplasts.

Like the early growth stages [43,54,56,84], the absence of functional TOC33 in *A. thaliana* results in a reduction in the chlorophyll content in *ppi1* when compared to the wild type (Figure 1). In contrast, *toc64-1* plants lacking a functional TOC64 do not show significant alterations in chlorophyll content (Figure 1). However, consistent with an impact of the function of the two proteins, TOC33 and TOC64, for chloroplast biogenesis [5,27], the photosynthetic performance and the non-photochemical quenching (NPQ) is reduced in both mutants compared to the wild type (Figure 1). The photosynthetic performance is not correlated to the ultrastructure for the thylakoids in the mutants, probably because the chloroplasts of *toc64-1* contained more and *ppi1* possessed fewer stacks per plastids compared to the wild type, while comparable disc numbers per stack were observed for all three genotypes (Figure 2).

**Figure 6:**
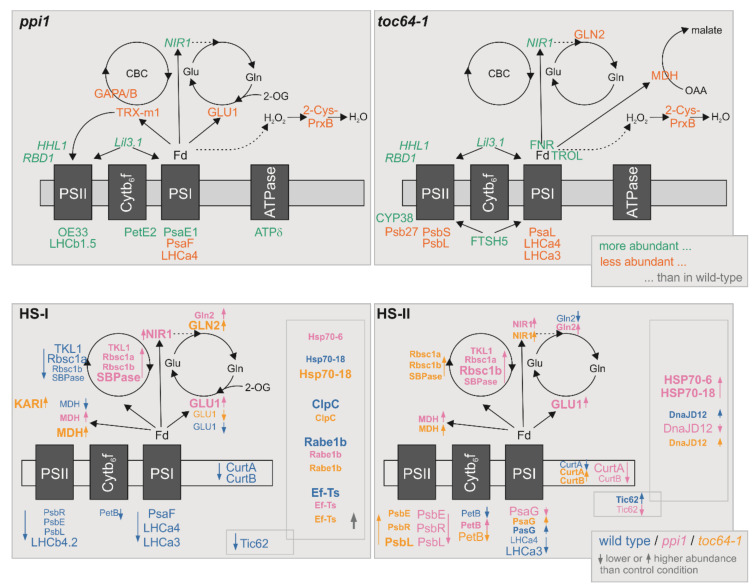
Proteomic alterations with respect to the genotype (top) or heat stress application (bottom). The results presented are summarized. On top, green indicates a higher and orange a lower abundance of the named protein in the indicated genotype (Fd: ferredoxin; CBC: Calvin Benson Cycle; for all other abbreviations and references see text). On the bottom, the color coding is according to the genotype (legend right bottom). Large letters indicate changes identified according to the described rule and small letters changes of at least two-fold. Normal letters indicated lower abundance (also indicated by downward arrow) and bold letters accumulation (upward arrow) when compared to the respective control sample. For further discussion, see text.

Even in early growth stages, the alteration of the chloroplast proteome in *ppi1* was rather moderate [84]. Comparing the previously identified proteins with either lower or higher abundance in 10-day-old *ppi1* seedlings with our results, we did not find the proteins enriched after ten days to be enriched at the later growth stage (Table 2); however, some of the proteins with lower abundance in 10-day-old *ppi1* plants were enriched in our analysis relative to the wild type (Table 2). Furthermore, we found only ten proteins with two-fold higher and eight proteins with two-fold lesser abundance in *ppi1* than the wild type (Figure 3; Table 1). The regulated proteins are centered around a reduced function of PSI or PSII, which is consistent with the reduced photosynthetic performance. Many proteins were identified to show higher abundance are either components of the photosystems (OE33, PsaE1, PsaF, LHCb1.5, LHCa4, PetE2) or involved in its assembly or protection (HHL1, RBD1, Lil3.1, Trx-m1; Figure 6 *ppi1*; [72,73,74,75,85]). The two exceptions are LHC4a and PsaF, which exhibit lower abundance in *ppi1* (Figure 3; Table 1). Remarkably, these two proteins interact in PSI [86], but the reason for the reduction in these two components of the LHC belt remains to be established. The others, mostly lower abundant proteins, are direct targets of ferredoxin (GLU1) or thioredoxins (GapA, GapB; Figure 6; [87,88]).

Summarizing, the mutation of the core TOC subunit in *ppi1* has a more substantial impact on the thylakoid architecture than *toc64-1*, but both mutants show reduced photosynthetic performance. The latter is consistent with the alteration of the proteomic content. However, while, in *ppi1* chloroplasts, mainly two PSI components exhibited lower abundance, in *toc64-1* chloroplasts, components of both PSI and PSII showed a lower abundance.

### 4.2. The Response of Chloroplasts to Temperature Elevations

Here, we tested the long-term memory to a single heat stress (HS) event (HS-I) and the adaptation to repetitive heat treatments (HS-II). Wild-type plants showed reduced photosynthetic performance after a single heat stress application (HS-I), but not after repetitive HS (HS-II, Figure 1). The same also holds for the chlorophyll content, which was reduced after HS-I but not after HS-II treatment. However, both treatments yielded a higher degree of NPQ, suggesting a degree of adaptation. Interestingly, the photosynthetic performance after HS-I in wild-type plants was comparable to that before and after the HS-I treatment of the mutant lines (Figure 1). The two mutant lines (*ppi1* and *toc64-1*) did not show a further reduction in the photosynthetic performance after HS-I, but an increase after repetitive heat stress application (HS-II; Figure 1). The NPQ was enhanced after both treatments. In *ppi1*, however, the chlorophyll content was higher when compared to the control conditions. On the other hand, HS treatment renders a similar NPQ in *toc64-1* after HS-I but not after HS-II. When compared to the wild type, the NPQ of *ppi1* was found to be lower under all conditions (Figure 1). Like the comparison of the chloroplast from different genotypes grown under control conditions, the changes of the photosynthetic performance after heat stress application do not correlate with the number of stacks per plastid and discs per stack (Figure 2).

TOC64 is required for chaperone association to the chloroplast surface [43,49,50,51,52]. In this context, HSP70 was enriched in the chloroplast fraction of *ppi1* plants and HSP81 in the wild type and *ppi1* after HS-II when compared to *toc64-1* under control conditions (Figure 4), while both these protein groups were highly abundant in chloroplasts of *toc64-1* after HS-I application. A specific displacement of chaperones could be masked by the transient nature of the interaction between TOC64 and HSPs and by HSP contaminations of the fraction, as chaperones are generally highly abundant in the cell. In turn, it is worth mentioning that tubulin-α and actin were enriched in chloroplasts of the wild type and *toc64-1* after HS-I, wherein actin was found to be enriched in the chloroplast fraction of *ppi1* only after HS-II (Figure 4). Whether this suggests a difference in chloroplast anchoring and movement that is discussed to involve actin [93] or stromule formation discussed to involved microtubules [94] remains to be investigated.

For the single HS (HS-I), we noticed a reduction in photosynthetic components and of enzymes involved in the Calvin Benson Cycle in the wild type when compared to the control conditions (Figure 5 and Figure 6, HS-I). This is consistent with the reduction in photosynthetic performance (Figure 1). Moreover, the number of stacks is reduced in wild-type plants, which coincides with a reduction in CurtA and CurtB described to act in thylakoid structure determination [76]. In contrast, the enzymes of the Calvin Benson Cycle showed higher abundance in the chloroplasts of *ppi1*, but not of *toc64-1* when compared to the respective control condition (Figure 5 and Figure 6, HS-I). Remarkably, class I Clp chaperones ClpC [81] were enriched in chloroplasts isolated from wild-type and *toc64-1*, and the translation elongation factor Rabe1b and its nucleotide exchange factor Ef-Ts known to be necessary for the HS response [82] were enhanced in all three genotypes (Figure 5 and Figure 6, HS-I). Moreover, HSP70-18 is enriched in chloroplasts from wild-type and *toc64-1* and HSP70-6 in *ppi1* (Figure 5 and Figure 6, HS-I). Thus, the long-term memory of a single heat application appears to be manifested by the upregulation of components of the chaperone and the translation system.

After repetitive HS application (HS-II), the chloroplast proteome of the wild type is somewhat comparable to the control condition. The only exceptions are two LHC components of PSI, which showed lower abundance (Figure 5 and Figure 6, HS-II). This is consistent with the comparable photosynthetic performance for plants grown under control or HS-II condition. In *ppi1* plants, we, again, noticed an accumulation of the enzymes of the Calvin Benson Cycle, but a reduction in PSI and PSII components when compared to control conditions (Figure 5 and Figure 6, HS-II). This is remarkable, as PsbL is required for PSII core dimers and PSII-LHCII complex formation [95], PsbR for PsbP assembly to PSII and PSII-LHCII complex formation [96] and PsbE for PSII and PsbB assembly [97,98]. Thus, its lower abundance in chloroplasts after HS-II when compared to control conditions stands somehow in contrast to the higher photosynthetic performance (Figure 1). In turn and consistent with the photosynthetic parameter, these proteins were enhanced in *toc64-1* (Figure 1,Figure 5 and Figure 6, HS-II). Alike in *ppi1*, enzymes of the Calvin Benson Cycle exhibited higher abundance in *toc64-1* chloroplasts after HS-II (Figure 5 and Figure 6, HS-II). As the PSII components, the two proteins CurtA and CurtB were reduced in *ppi1* but enhanced in *toc64-1*, which coincides with the alteration of the disc number per thylakoid stack (Figure 2, Figure 5 and Figure 6, HS-II). For the alteration of the abundance of regulatory components, HSP70-18 and HSP70-6 were found to accumulate in *ppi1* only, while a protein assigned as DnaJ D12 showed low abundance in *ppi1* but higher abundance in the other two genotypes (Figure 5 and Figure 6, HS-II). This is in contrast to the HS-I treatment; thus, it appears that the chloroplasts did not manifest a memory system but have adapted to the new situation on exposure to repetitive heat stress application (HS-II).

## Figures and Tables

**Table 1:** Proteins predicted to be plastidic localized and exhibiting lower or higher abundance in chloroplasts isolated from mutants in comparison to wild-type chloroplasts. The protein name, the process the protein is acting in, the accession number and the obtained label-free quantification (LFQ) values (log_2_) are shown. The fold change (FC) according to the LFQ values, is presented for *toc64-1* and *ppi1* in comparison to the wild type; fold-change values >2 or <2 are marked in green or red, respectively.

Name	Process	Accession	log_2_(LFQ)			FC (LFQ)	
			wt	*toc64-1*	*ppi1*	*toc64-1*	*ppi1*
CYP38	photosynthesis	AT3G01480	20.44	21.96	21.10	2.87	1.59
FNR2	photosynthesis	AT1G20020	21.98	23.13	22.91	2.22	1.91
cpHSP70-1	protein synth.	AT4G24280	19.71	20.82	19.92	2.16	1.16
TROL	photosynthesis	AT4G01050	21.57	22.67	22.45	2.14	1.84
Rpl15	protein synth.	AT3G25920	18.01	19.08	19.07	2.10	2.08
FTSH5	photosynthesis	AT5G42270	22.11	23.12	22.93	2.02	1.77
LHCb1.5	photosynthesis	AT2G34420	21.34	22.11	23.95	1.71	6.11
Tic62	TIC	AT3G18890	21.27	22.22	22.58	1.93	2.48
LOX2	JA synth.	AT3G45140	21.09	21.20	22.24	1.07	2.22
unknown		AT3G61870	19.60	20.08	20.75	1.40	2.22
PetE2	photosynthesis	AT1G20340	22.12	22.11	23.27	0.99	2.21
PsaE1	photosynthesis	AT4G28750	23.61	24.14	24.70	1.45	2.14
cpATPδ	photosynthesis	AT4G09650	21.75	22.17	22.84	1.34	2.14
OE33	photosynthesis	AT3G50820	22.15	23.10	23.21	1.92	2.08
unknown		AT5G37360	18.59	18.78	19.61	1.14	2.03
LHCa4	photosynthesis	AT3G47470	24.35	23.16	23.33	0.44	0.49
*GAPb*	metabolism	AT1G42970	21.99	21.46	20.89	0.69	0.47
*PrxB*	regulation	AT5G06290	21.53	20.19	20.40	0.39	0.46
*PsaF*	photosynthesis	AT1G31330	25.84	24.89	24.68	0.52	0.45
Ef-Ts	protein synth.	AT4G29060	19.02	19.13	17.82	1.08	0.44
*Trx-M1*	regulation	AT1G03680	19.54	18.58	18.30	0.52	0.43
*GAPa*	metabolism	AT3G26650	23.48	22.94	22.23	0.68	0.42
*GLU1*	metabolism	AT5G04140	20.81	20.40	19.38	0.75	0.37
*Psb27*	photosynthesis	AT1G03600	22.30	21.26	22.32	0.49	1.01
*PsbS*	photosynthesis	AT1G44575	24.27	23.22	23.53	0.48	0.60
*PsaL*	photosynthesis	AT4G12800	24.04	22.92	23.32	0.46	0.61
*GLN2*	metabolism	AT5G35630	22.26	21.14	21.62	0.46	0.64
*NADP-MDH*	metabolism	AT5G58330	18.95	17.75	18.47	0.44	0.72
LHCa3	photosynthesis	AT1G61520	25.19	23.94	24.19	0.42	0.50
*PsbL*	photosynthesis	ATCG00560	21.12	16.75	20.91	0.05	0.86

**Table 2:** Comparison of protein changes in chloroplasts isolated from young (10 days; [84]) and older plants (21 days; present study). Given are the name, the accession number, the reported fold change for chloroplasts from 10-day old plants (*ppi1*/wild-type), the LFQ (log_2_) observed here for the same proteins and the fold change of the LFQ for *toc64-1* and *ppi1* when compared to the wild type (wt). n.d.: not detected. Fold-change values >1.5 are marked in red.

Name	Accession	FC [84]	log_2_(LFQ)			FC (LFQ)	
			wt	*toc64-1*	*ppi1*	*toc64-1*	*ppi1*
HSP90 homolog	At2g04030	2.4/1.8	19.96	n.d.	19.97	n.d.	1.00
Chaperonin 60β	At1g55490	1.9/1.6	21.92	22.56	22.09	1.57	1.13
	At3g13470		n.d.	n.d.	n.d.	n.d.	n.d.
HSP70 homolog	At4g24280	1.8	19.71	20.82	19.92	2.16	1.16
	At5g49910		n.d.	n.d.	n.d.	n.d.	n.d.
PPIase	At3g62030	1.8	21.06	20.67	20.51	0.77	0.68
EF-Tu homolog	At4g20360	1.7	22.74	22.33	22.58	0.75	0.90
Chaperonin 60α	At2g28000	1.6	21.59	22.03	21.89	1.36	1.23
R-5-P isomerase	At3g04790	0.6/0.5	19.37	n.d.	19.91	n.d.	1.45
*LHCb5*	At4g10340	0.6	25.82	25.65	26.30	0.89	1.39
LHCII type 1	At1g29910	0.6	25.85	25.58	26.66	0.83	1.74
	At1g29920						
	At1g29930						
	At2g34420		21.34	22.12	23.95	1.72	6.11
OE23	At1g06680	0.6	24.82	25.27	25.19	1.37	1.29
OE33	At3g50820	0.6/0.5	22.15	23.11	23.21	1.93	2.08
	At5g66570		25.03	25.66	25.88	1.55	1.79
*SSU*	At1g67090	0.4	23.62	23.37	23.48	0.85	0.91

The proteome of chloroplasts isolated from *toc64-1* plants shows distinct alterations when compared to the wild type and *ppi1* (Figure 3 and Figure 6; *toc64-1* panel). The observed changes in protein abundance again are centered around PSI or PSII, which is consistent with the reduced photosynthetic performance of the mutant. However, we did not find PS components, but many regulatory factors with higher abundance (HHL1, RBD1, Lil3.1, Trx-m1, Cyp38, FNR, TROL, FTSH5; Figure 6; [72,73,74,75,85,89,90,91,92]). In *toc64-1*, the five PS components with altered abundance are all reduced (Figure 3; Table 1). As for *ppi1*, the other mostly downregulated proteins are linked to the action of ferredoxin and FNR: GLN2, MDH, TRX-m1 and PrxB (Figure 6).

## Supplementary Materials

The following are available online at https://www.mdpi.com/2073-4425/11/6/650/s1. Table S1: Protein Group output from MaxQuant software for the proteome analysis. The table contains information on the identified protein groups (multiple entries that could not be differentiated based on peptide evidence), the majority protein ID (the leading protein of the protein group; note, in case that multiple majority IDs were identified for one group, each of them is listed separately), the FASTA information for the majority protein, the number of peptides for the majority protein in total and in each experimental application and the identification type, the intensity observed, the LFQ intensity observed, Table S2: Specific information on identified proteins. Given is the majority protein ID; the protein IDs of the proteins found in the same group and the SUBA based localization showing the subcellular localization with the highest confidence score. Subsequently, the FASTA headers/function defines the identified or predicted cellular or molecular function of identified proteins. Finally, the identification mode by which proteins were identified per sample, either by MS/MS or by chromatogram alignment with other samples in which the proteins were directly identified is indicated. Note, all columns except SUBA based localization were extracted from Table S1, and the FASTA function is limited to essential information, Supplemental file S1: R scripts for the statistical analysis performed on Table S1 using R 4.0.0 and the DEP package.
