# Supplementary material for: Structural and Functional Heat Stress Responses of Chloroplasts of Arabidopsis thaliana
Source: Genes (Basel). 2020 Jun 12;11(6):650. doi: 10.3390/genes11060650 (PMC7349189; doi:10.3390/genes11060650)
Supplement: Supplementary file 1 [file genes-11-00650-s001.zip › Supplements/Supplemental File S1.pdf]

R scripts for “Structural and functional heat stress responses of chloroplasts of Arabidopsis thaliana”

Maik Böhmer

2020-06-03

```
R version 4.0.0 (2020-04-24)
Platform: x86_64-w64-mingw32/x64 (64-bit)
Running under: Windows 10 x64 (build 18363)

attached packages:
ggplot2_3.3.1
reshape2_1.4.4
dplyr_1.0.0
DEP_1.10.0
```

Table 1 script

```
library("DEP")
library("dplyr")

##
## Attaching package: 'dplyr'

## The following objects are masked from 'package:stats':
##
##     filter, lag

## The following objects are masked from 'package:base':
##
##     intersect, setdiff, setequal, union

setwd("F:/Enrico/")

# data import
data <- read.delim("F:/Enrico/combined/txt/proteinGroups.txt")
expdesign <- read.delim("F:/Enrico/Experiment_design_notreat.txt",
stringsAsFactors = FALSE)

# We filter for contaminant proteins and decoy database hits, which are
indicated by "+" in the columns "Potential.contaminants" and "Reverse",
respectively.
data <- filter(data, Reverse != "+", Potential.contaminant != "+",
Only.identified.by.site != "+")

# Make unique names using the annotation in the "Gene.names" column as
primary names and the annotation in "Protein.IDs" as name for those that do
not have an gene name.
```

```r
data_unique <- DEP::make_unique(data, "Fasta.headers", "Protein.IDs", delim =
";")

# Generate a SummarizedExperiment object by parsing condition information
from the column names
LFQ_columns <- grep("LFQ.", colnames(data_unique)) # get LFQ column numbers
data_se <- make_se(data_unique, LFQ_columns, expdesign)

# filter for presence in all conditions
data_filt <- filter_proteins(data_se, type = "complete")

# turn se object into dataframe
wide <- get_df_wide(data_filt)
wide <- wide[1:5]

#calculate FC values for toc64 and ppi
wide$FC_toc64 <- 2 ^ (wide$toc64.ctl_1 - wide$wt.ctl_1)
wide$FC_ppi <- 2 ^ (wide$ppi.ctl_1 - wide$wt.ctl_1)

# Filter for proteins with differential absolute abundance >2
table1 <- wide[which(wide$FC_toc64 >2| wide$FC_ppi >2 | wide$FC_toc64 <0.5 |
wide$FC_ppi <0.5), ]
```

Table 2 script

```r
library("DEP")
library("dplyr")

##
## Attaching package: 'dplyr'

## The following objects are masked from 'package:stats':
##
##     filter, lag

## The following objects are masked from 'package:base':
##
##     intersect, setdiff, setequal, union

library(reshape2)
library(grid)
library(reshape)

##
## Attaching package: 'reshape'

## The following objects are masked from 'package:reshape2':
##
##     colsplit, melt, recast
```

```
## The following object is masked from 'package:dplyr':
##
##     rename

library(ggplot2)

setwd("f:/Enrico/")

# data import
data <- read.delim("F:/Enrico/combined/txt/proteinGroups.txt")
expdesign <- read.delim("F:/Enrico/Experiment_design.txt", stringsAsFactors =
FALSE)

# We filter for contaminant proteins and decoy database hits, which are
indicated by "+" in the columns "Potential.contaminants" and "Reverse",
respectively.
data <- filter(data, Reverse != "+", Potential.contaminant != "+",
Only.identified.by.site != "+")

# Make unique names using the annotation in the "Gene.names" column as
primary names and the annotation in "Protein.IDs" as name for those that do
not have an gene name.
data_unique <- DEP::make_unique(data, "Fasta.headers", "Protein.IDs", delim =
";")

# Generate a SummarizedExperiment object by parsing condition information
from the column names
LFQ_columns <- grep("LFQ.", colnames(data_unique)) # get LFQ column numbers
data_se <- make_se(data_unique, LFQ_columns, expdesign)

# filter for presence in at least 5 conditions
data_filt <- filter_proteins(data_se, type = "fraction", min = 0.5)

# turn se object into dataframe
wide <- get_df_wide(data_filt)
wide <- wide[c(11,1,2:10)]

#normalize to minimum value
wide$min <- do.call(pmin, c(wide[3:11], na.rm = TRUE))
wide.norm <- wide[,3:11] - wide[,12]
wide.norm <- merge(wide[,1:2],wide.norm[,1:9])

#calculate FC values for toc64 and ppi
wide$FC_toc64 <- 2 ^ (wide$toc64.ctl_1 - wide$wt.ctl_1)
wide$FC_ppi <- 2 ^ (wide$ppi.ctl_1 - wide$wt.ctl_1)

# generate table 2
table2 <- wide[,c(1:5,13:14)]
patterns <- c("AT2G04030",
                  "AT1G55490",
```

```
                  "AT3G13470",
                  "AT4G24280",
                  "AT5G49910",
                  "AT3G62030",
                  "AT4G20360",
                  "AT2G28000",
                  "AT3G04790",
                  "AT4G10340",
                  "AT1G29910",
                  "AT1G29920",
                  "AT1G29930",
                  "AT2G34420",
                  "AT1G06680",
                  "AT3G50820",
                  "AT5G66570",
                  "AT1G67090"
)
table2 <- table2[grep(paste(patterns, collapse="|"),
table2[["Protein.IDs"]]), ]
```

Figure 5 data script

```
#calculate FC values for HSI and HSII
wide$FC_toc64_HSI <- 2 ^ (wide$toc64.HSI_1 - wide$toc64.ctl_1)
wide$FC_toc64_HSII <- 2 ^ (wide$toc64.HSII_1 - wide$toc64.ctl_1)
wide$FC_ppi_HSI <- 2 ^ (wide$ppi.HSI_1 - wide$ppi.ctl_1)
wide$FC_ppi_HSII <- 2 ^ (wide$ppi.HSII_1 - wide$ppi.ctl_1)
wide$FC_wt_HSI <- 2 ^ (wide$wt.HSI_1 - wide$wt.ctl_1)
wide$FC_wt_HSII <- 2 ^ (wide$wt.HSII_1 - wide$wt.ctl_1)

# significant changes (> 1.65)
wide.sig <- subset(wide, wide[, 15:20] > 1.65 | wide[, 15:20] < 0.61)
wide.sig <- na.omit(wide)
```
